# Efficacy comparison between intramedullary nail fixation and plate fixation in distal tibia fractures: a meta-analysis of randomized controlled trials

**DOI:** 10.1186/s13018-024-04900-y

**Published:** 2024-07-12

**Authors:** Xiaobin Li, Kaipeng Chen, Haipeng Xue, Junwen Cheng, Xiaoping Yu

**Affiliations:** 1 Department of Traumatic Orthopedics, Yuncheng Central Hospital affiliated to Shanxi Medical University, Yuncheng, Shanxi Province 044000 China; 2 Department of Clinical Laboratory, Yuncheng Central Hospital affiliated to Shanxi Medical University, No. 3690, Hedong East Street, Yanhu District, Yuncheng, Shanxi Province 044000 China

**Keywords:** Distal tibia fractures, Intramedullary nail fixation, Plate fixation, Infection, Complication, Meta-analysis

## Abstract

**Background:**

Intramedullary nail (IMN) and plate fixation are the most commonly used surgical modalities for distal tibia fractures. However, the superiority of their efficacy regarding functional outcomes and complications remains controversial. Here, we performed a systematic review and meta-analysis to compare the efficacy of these two modalities.

**Methods:**

Randomized controlled trials (RCTs) comparing the efficacy of IMN and plate fixation in distal tibia fractures were searched in PubMed, Web of Science, EMBASE, ClinicalTrials.gov, and Cochrane Library up to January 31, 2024. Weighted mean difference (WMD) and odds ratio (OR) with corresponding 95% confidence interval (CI) were estimated using a random-effect model for continuous and categorical outcomes, respectively.

**Results:**

A total of 20 RCTs comprising 1528 patients were included. Compared with plate fixation, IMN significantly shortened surgery time (WMD=-10.73 min, 95%CI: -15.93 to -5.52), union time (WMD=-1.56 weeks, 95%CI: -2.82 to -0.30), and partial (WMD=-1.71 weeks, 95%CI: -1.91 to -0.43) and full (WMD=-2.61 weeks, 95%CI: -3.53 to -1.70) weight-bearing time. IMN was associated with markedly reduced risk of wound infection (OR = 0.44, 95%CI: 0.31–0.63) and secondary procedures (OR = 0.72, 95%CI: 0.55–0.95), but increased the risk of malunion (OR = 1.53, 95%CI: 1.02–2.30) and anterior knee pain (OR = 3.94, 95%CI: 1.68–9.28). The rates of nonunion, delayed union, and functional assessment scores did not significantly differ between the two groups. The percentages of patients obtaining an excellent functional outcome or an excellent and good functional outcome post-operation were comparable.

**Conclusions:**

Both IMN and plate fixation are effective modalities for the surgical treatment of distal tibia fractures. IMN seems to be preferred since it confers more advantages, but the elevated rates of malunion and knee pain require attention. The decision on fixation modality should be tailored to the specific fracture, considering these pros and cons.

**Supplementary Information:**

The online version contains supplementary material available at 10.1186/s13018-024-04900-y.

## Background

The tibia, attached proximally to the femur at the knee and distally to the ankle, is one of most important weight-bearing bones in the lower limb. Distal tibia fractures, resulting from h high-energy and low-energy trauma, are common in traffic accidents and athletic injuries, accounting for 37.8% of all tibia fractures [1]. Managing distal tibia fractures is challenging due to their unique anatomical characteristics, such as proximity to the skin at the anterior medial side with low soft tissue coverage and poor blood supply [2]. It is adjacent to the ankle joint and only provides a narrow segment for hardware implant. These fracture are usually associated with nonunion, malunion, wound infections, and wound dehiscence [3].

Currently, the most commonly used treatment modalities of distal tibia fractures are intramedullary nail (IMN) and plate fixation [4]. IMN is minimally invasive, involves small skin incisions, allows for easy removal of nails, preserves more extraosseous blood supply, and causes less soft tissue damage [5]. It facilitates earlier union and weight-bearing and reduces the risk of various complications [6–9]. However, anterior knee pain and malunion are significant concerns [10]. Plate fixation is implemented through open reduction and internal fixation (ORIF) or minimally invasive percutaneous plate osteosynthesis (MIPO). ORIF method involves more extensive wound exposure and soft tissue dissection, leading to higher risks of wound complications and delayed union [11]. Whereas, MIPO employs an indirect bridge reduction technique and causes less damage to blood supply, potentially mitigating ORIF drawbacks [12, 13].

There is no consensus on the superiority of IMN or plate fixation for distal tibia fractures, making the choice of optimal surgical modality debatable. Numerous retrospective studies comparing both surgical methods provide low evidence levels due to their design [14–16]. Several randomized controlled trials (RCTs) have focused on this issue but often have small sample sizes, limiting their statistical power to detect differences in complication rates [17–19]. Aiming to provide high levels of evidence for surgical decision-making in distal tibia fractures, we performed a systematic review and meta-analysis of RCTs to compare the efficacy of IMN and plate fixation regarding functional outcomes and complications.

## Methods

### Search strategy

This systematic review and meta-analysis was conducted following the Preferred Reporting Items for Systematic review and Meta-Analysis (PRISMA) guideline (Additional file 1) [20]. RCTs comparing intramedullary nail fixation to plate fixation in extra-articular distal tibia fractures were searched in PubMed, Web of Science, EMBASE, Clinicaltrials.gov, and the Cochrane Library. The last search date was January 31, 2024. The literature search used MeSH terms and their variations, with search strategies listed in Additional file 2: Table S1. There was no language restrictions. Reference lists of identified articles and relevant reviews were also manually searched.

### Inclusion and exclusion criteria

Article eligibility was judged according to the PICOS framework. Participants (P): Adult patients aged ≥ 16 years with extra-articular distal tibia fractures caused by trauma. Intervention (I): IMN fixation. Comparison (C): Plate fixation. Outcomes (O): Surgical efficacy, including functional outcomes and complications. Study design (S): RCTs. Fractures related to osteoporosis, tumors, and systematic diseases were excluded. Non-randomized observational studies, case series, conference abstracts, reviews, meta-analyses, and biomechanical studies were discarded. Trials with less than 6 months of follow-up were also excluded.

### Outcome measurement

Comparisons of surgical efficacy included surgery time, radiation time, union time, partial weight-bearing time, full weight-bearing time, wound infection, union complications (nonunion, delayed union, malunion), anterior knee pain, secondary procedures, and functional assessment. Secondary procedures included debridement, revision, implant removal, and bone grafting. Functional assessment used various scoring systems, such as the American Orthopaedic Foot and Ankle surgery (AOFAS) scoring system [21], Disability Rating Index (DRI) [22], Foot Function Index (FFI) [23], and Olreud Molander Ankle Scores (OMAS) [24], Mazur ankle score (MAS) [25]. Functional outcomes were rated as an excellent, good, fair, or poor based on scores.

### Methodology assessment

Two independent reviewers (XL, KC) assessed methodology using the Cochrane Collaboration’s tool for assessing risk of bias [26]. The risk of bias was graded as low, unclear, and high in domains of random sequence generation, allocation concealment, blinding of participants and personnel, blinding of outcome assessment, incomplete outcome, selective reporting, and other bias. Disagreements were resolved through discussion by a third researcher (XP).

### Data extraction

Extracted information included the first author, publication year, country, fracture type (closed, open), OTA/AO classifications, plate fixation method (MIPO, ORIF), sample size, mean age, percentages of males and fibula fractures, follow-up duration, and quantitative data of aforementioned outcome measurements. Literature search, selection, and data extraction were performed by two independent reviewers (KC, HX). Disagreements were resolved through discussion or a third researcher (JC).

### Statistical analysis

Meta-analysis was conducted using STATA 16.0 (StataCorp, TX, USA). Heterogeneity was assessed by I^2^ statistic and Q test. Given the diversity in patients, fractures, and surgical procedures among the included studies, we applied a random-effect model for all pooled analyses to gain a more conservative results regardless of heterogeneity. For continuous outcomes, the weighted mean difference (WMD) between IMN and plate fixation groups was calculated with corresponding 95% confidence interval (95%CI). For dichotomous outcomes, the odds ratio (OR) with 95%CI was calculated. Higher scores in AOFAS, OMAS and MAS indicated better function, while higher scores in DRI and FFI represented greater impairment; thus, they were separately analyzed. Subgroup analysis stratified by plate fixation technique (MIPO, ORIF), AO classification (AO 42, AO 43), and inclusion of open fractures was conducted. Sensitivity analysis by sequentially omitting one study was done to evaluate the robustness of the meta-analysis. Meta-regression analysis was conducted to explore whether baseline characteristics were associated with the effect size of pooled analysis if ten or more studies were available. Funnel plot symmetry was viewed and Egger’s test was conducted for publication bias. A trim-and-fill analysis, including imputed studies, was implemented if potential publication bias was suggested (Egger’s test *P* < 0.10). P value less than 0.05 was considered statistically significant.

## Results

### Baseline features of studies included in meta-analysis

**Fig. 1:**
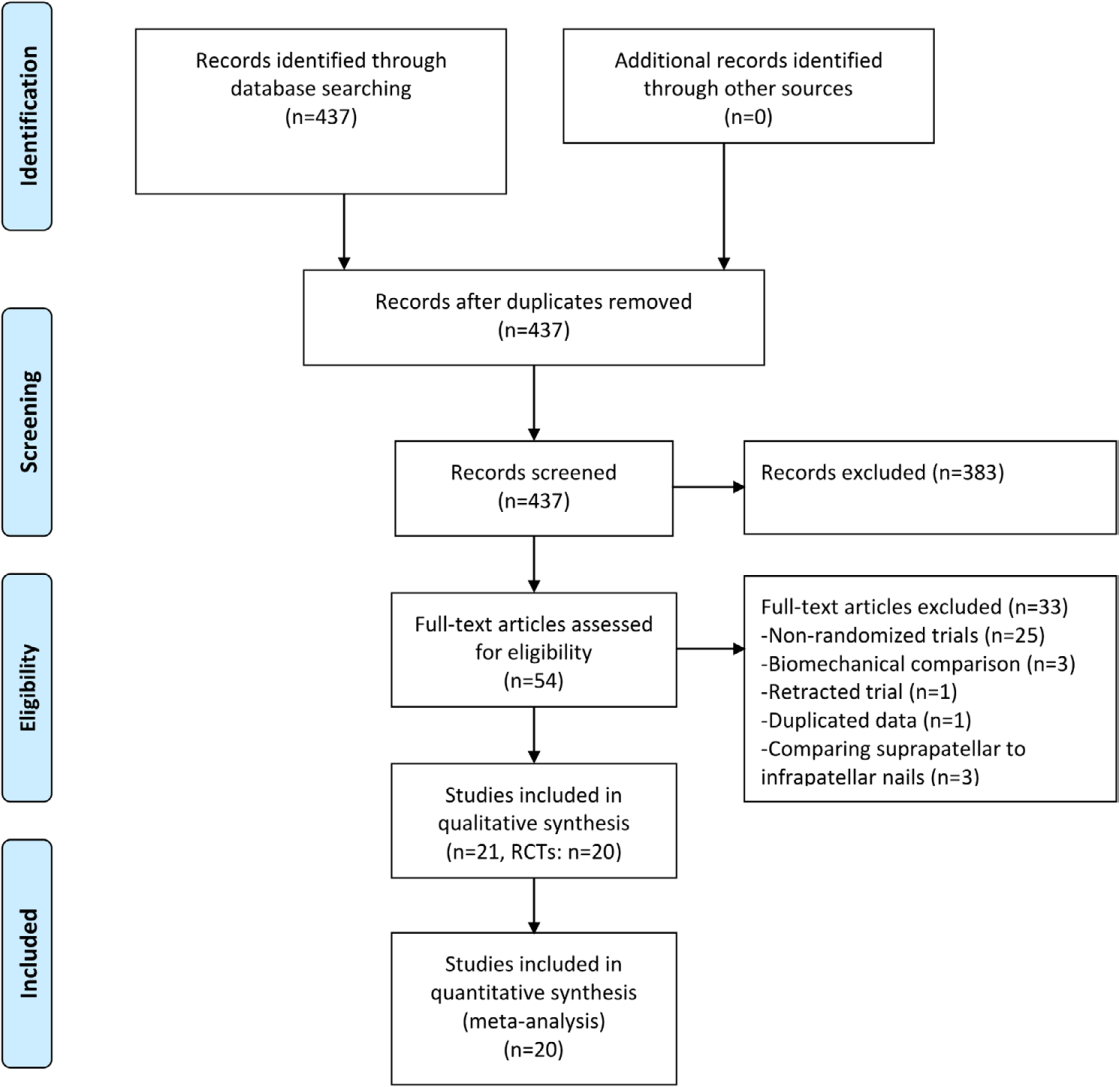
Flowchart of literature search and selection

Among the 437 retrieved articles, 383 were discarded after reviewing titles and abstracts. The remaining 54 articles were reviewed for full texts, and 33 were excluded for the following reasons: non-randomized trials (*n* = 25), biomechanical comparative studies (*n* = 3), duplicated data (*n* = 1), retracted study (*n* = 1), and comparison of suprapatellar and infrapatellar IMN (*n* = 3). Finally, 21 eligible studies were included in the meta-analysis (Fig. 1) [17–19, 27–44]. Two studies reported different outcomes from the same RCT and were merged [32, 33]. A total of 1528 participants were analyzed: 765 patients in the IMN group and 763 in the plate fixation group. The sample size ranged from 24 to 321, with only four studies exceeding 100 participants [27, 32, 34, 35]. Thirteen trials only recruited closed fractures, whereas open fractures were not excluded in the other seven trials. The majority of included studies applied plate fixation via the MIPO technique, three used the ORIF technique [29, 32, 43], and one used both techniques [37]. All studies had at least one year of follow-up except for three trials [39, 41, 44]. Fifteen studies reported the distributions of AO 42 or AO 43 classification in both groups, while the remaining five did not report the distributions [27, 31, 38, 41, 44]. The baseline features of all trials were summarized in Table 1, and the characteristics of fractures were shown in Table 2.

**Table 1:** Baseline characteristics of studies included in meta-analysis

Study	Country	Fracture type	No.^#^	Male^#^	Mean age, years^#^	Plating approach	Follow-up, months
Im, 2005	Korea	Closed or open G1	34/30	22/24	42 (range:19–65)/40 (range:17–60)	ORIF	≥ 24
Guo, 2010	China	Closed	44/41	26/24	44.2 (range 27–70)/44.4 (range: 23–69)	MIPO	≥ 12
Vallier, 2011	USA	Closed or open G1-3	56/48	45/40	38.1/38.5	ORIF	19.9
Mauffrey, 2012	UK	Closed or open G1	12/12	7/9	Median 50 (IQR: 39–60)/ 33 (IQR:24–43)	MIPO	≥ 12
Li, 2014	China	Closed or open G1-2	46/46	41/38	44 (range:18–78)/43 (range:18–79)	MIPO	14.8
Vaza, 2014	India	Closed or open G1	20/20	NR	Total range: 18–65	Mixed	≥ 12
Polat, 2015	Turkey	Closed	10/15	9/7	34.0 ± 9.7/36.4 ± 10.7	MIPO	23.1
Fang, 2016	China	Closed or open G1-2	28/28	19/21	35.0 ± 9.2/38.6 ± 7.5	MIPO	28.9
Daolagupu, 2017	India	Closed	21/21	17/15	35.19 ± 9.22/39.09 ± 10.13	MIPO	≥ 12
Costa, 2017	UK	Closed	161/160	96/101	44.3 ± 16.3/45.8 ± 16.2	MIPO	≥ 12
Wani, 2017	India	Closed	30/30	22/20	36.4 ± 9.7/38.4 ± 8.7	MIPO	≥ 12
Rabari, 2017	India	Closed	30/43	27/38	41.2 ± 10.28/39.98 ± 9.64	MIPO	≥ 12
Ali, 2017	India	Closed or open G1	30/30	23/21	40.4 ± 12.3/41.9 ± 13.5	MIPO	≥ 9
Prasad, 2017	India	Closed	15/15	12/13	35.67 ± 8.12/36.57 ± 7.68	MIPO	≥ 12
Basit, 2019	Pakistan	Closed	12/12	11/12	31 (range:22–35)/22.5 (range:20-34.5)	MIPO	≥ 9
Kariya, 2020	India	Closed	73/69	44/43	43.7 ± 15.3/45 ± 14.4	MIPO	34.8
Lakhotia, 2020	India	Closed	25/25	17/16	44.4 ± 14.11/41.96 ± 15.81	MIPO	28.1
Keerio, 2021	Pakistan	Closed	30/30	Total 42	41.5/38.2	ORIF	≥ 12
Kc, 2022	Neapl	Closed	50/50	26/28	37.34 ± 11.21/41.14 ± 10.52	MIPO	≥ 12
Haider, 2022	Pakistan	Closed	38/38	22/20	30.8 ± 6.42/31.1 ± 3.58	MIPO	≥ 8

^#^ Intramedullary nailing group/plate fixation group. IQR: interquartile range; MIPO: minimally invasive plate osteosynthesis. NR: not reported; ORIF: open reduction and internal fixation

**Table 2:** Characteristics of fractures in meta-analysis

Study	AO 42 classification			AO 43 classification			Closed/open fractures			Fibula fractures		Distance^#^
	IMN	Plate		IMN	Plate		IMN	Plate		IMN	Plate	
Im, 2005	NR	NR		A1 (15), A2 (9), A3 (5), C1 (5)	A1 (11), A2 (10), A3 (4), C1 (5)		26/8	25/5		22	15	NR
Guo, 2010	NR	NR		A1 (13), A2 (16), A3 (15)	A1 (13), A2 (12), A3 (16)		44/0	41/0		NR	NR	≥ 3 cm
Vallier, 2011	A (32), B (17), C (7)	A (31), B (10), C (7)		NR	NR		35/21	29/19		Total: 28		4–11 cm
Mauffrey, 2012	NR	NR		12	12		NR	NR		NR	NR	≤ 2 Muller squares
Li, 2014	A (33), B (8), C (5)	A (37), B (7), C (2)		NR	NR		29/17	32/14		NR	NR	4–11 cm
Vaza, 2014	NR	NR		A1 (5), A2 (10), A3 (3), B1 (2)	A1 (4), A2 (8), A3 (5), B1 (3)		13/7	15/5		18	17	≤ 5 cm
Polat, 2015	A1 (6), A2 (3), A3 (1)	A1 (11), A2 (1), A3 (3)		NR	NR		10/0	15/0		8	6	4–12 cm
Fang, 2016	A (16), B (8), C (4)	A (15), B (10), C (3)		NR	NR		24/4	22/6		20	19	6–12 cm
Daolagupu, 2017	NR	NR		A1 (11), A2 (6), A3 (4)	A1 (10), A2 (9), A3 (2)		21/0	21/0		Total: 37		≥ 3 cm
Costa, 2017	NR	NR		NR	NR		161/0	160/0		NR	NR	≤ 2 Muller squares
Wani, 2017	A1 (18), A2 (9), A3 (3)	A1 (20), A2 (2), A3 (8)		NR	NR		30/0	30/0		6	8	NR
Rabari, 2017	NR	NR		30	43		30/0	43/0		NR	NR	NR
Ali, 2017	A (17), B (6), C (0)	A (16), B (5), C (1)		A (7)	A (8)		20/10	23/7		NR	NR	NR
Prasad, 2017	NR	NR		A1 (8), A2 (5), A3 (2)	A1 (9), A2 (4), A3 (2)		15/0	15/0		NR	NR	≤ 5 cm
Basit, 2019	NR	NR		12	12		12/0	12/0		NR	NR	≤ 6 cm
Kariya, 2020	A2.1 (55), A2.2 (13), A2.3 (5)	A2.1 (51), A2.2 (15), A2.3 (3)		NR	NR		73/0	69/0		73	69	3–12 cm
Lakhotia, 2020	NR	NR		A1 (10), A2 (11), A3 (4)	A1 (12), A2 (18), A3 (5)		25/0	25/0		20	22	NR
Keerio, 2021	NR	NR		A1 (11), A2 (10), A3 (9)	A1 (13), A2 (12), A3 (5)		30/0	30/0		NR	NR	NR
Kc, 2022	NR	NR		A1 (38), A2 (11), A3 (1)	A1 (36), A2 (12), A3 (2)		50/0	50/0		NR	NR	≥ 3 cm
Haider, 2022	NR	NR		38	38		38/0	38/0		NR	NR	NR

^#^ Distance of fractures from the ankle joint. IMN: intramedullary nailing; NR: not reported

### Methodology assessment

The risk of bias assessment was summarized in Additional file 2: Table S2. Most trials applied appropriate randomization procedures, but less than half reported allocation concealment. Since it was impossible to blind patients and surgeons, the performance bias (blinding of participants and personnel) of all trials was deemed unknown risk. In four trials, the outcome assessment was performed by independent researchers who were not involved in operation and post-operation management [27, 28, 31, 41]. Thus, these trials were considered to have low risk of detection bias, while the others had unknown risk. One trial had a high percentage of loss to follow-up and therefore had high risk of attrition bias [42].

### Surgery time, radiation time, union time and weight-bearing time

**Fig. 2:**
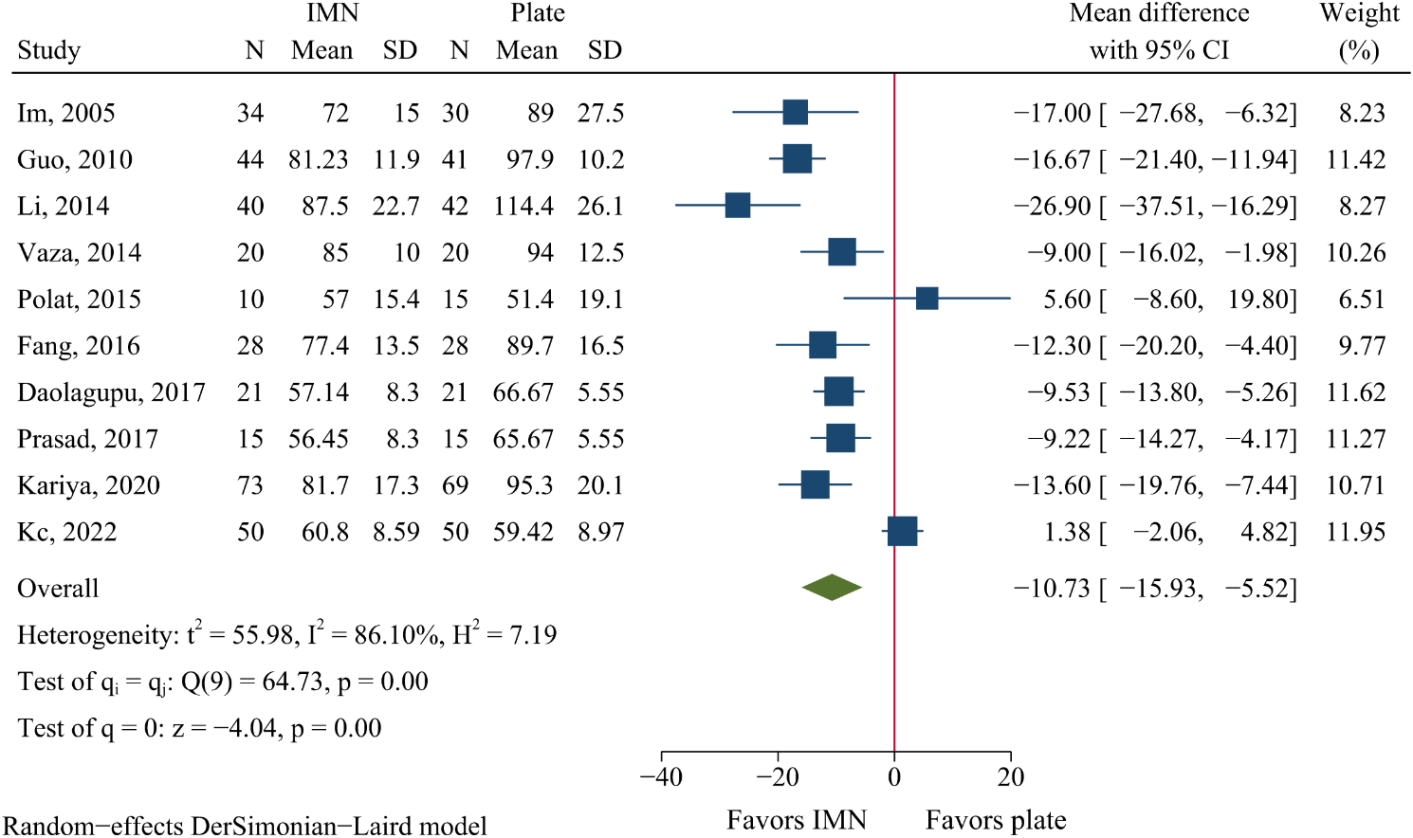
Meta-analysis of surgery time in minutes comparing nail to plate fixation

Ten studies comprising 666 patients compared surgery time between IMN and plate fixation groups. IMN had significantly shorter surgery time than plate fixation (WMD=-10.73 min, 95%CI: -15.93 to -5.52, *P* < 0.001, Fig. 2).

Radiation time was compared between both surgical modalities in five trials involving 408 fractures. Pooled analysis showed significantly shortened radiation time in the IMN group compared to the plate group (WMD=-0.79 min, 95%CI: -1.31 to -0.27, *P* = 0.003; Additional file 3: Figure S1).

**Fig. 3:**
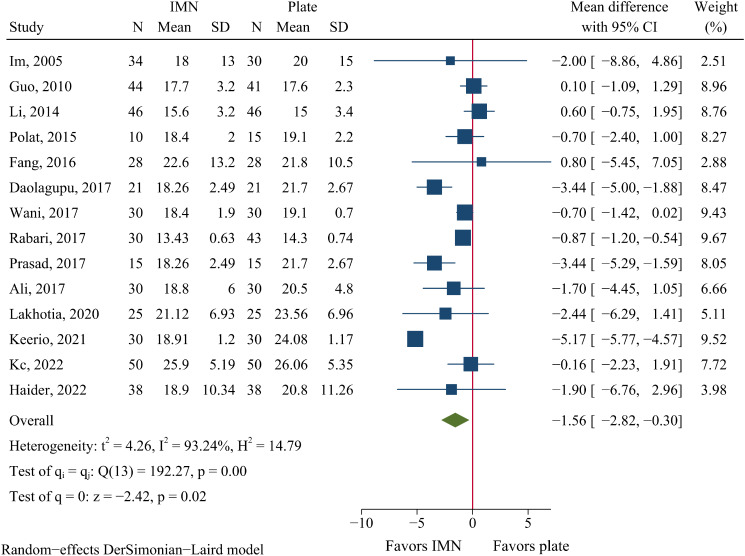
Meta-analysis of union time in weeks comparing nail to plate fixation

Union time was reported in 14 trials including 873 fractures. We found that patients treated with IMN achieved union earlier than those treated with plate fixation (WMD=-1.56 weeks, 95%CI: -2.82 to -0.30, *P* = 0.015, Fig. 3).

**Fig. 4:**
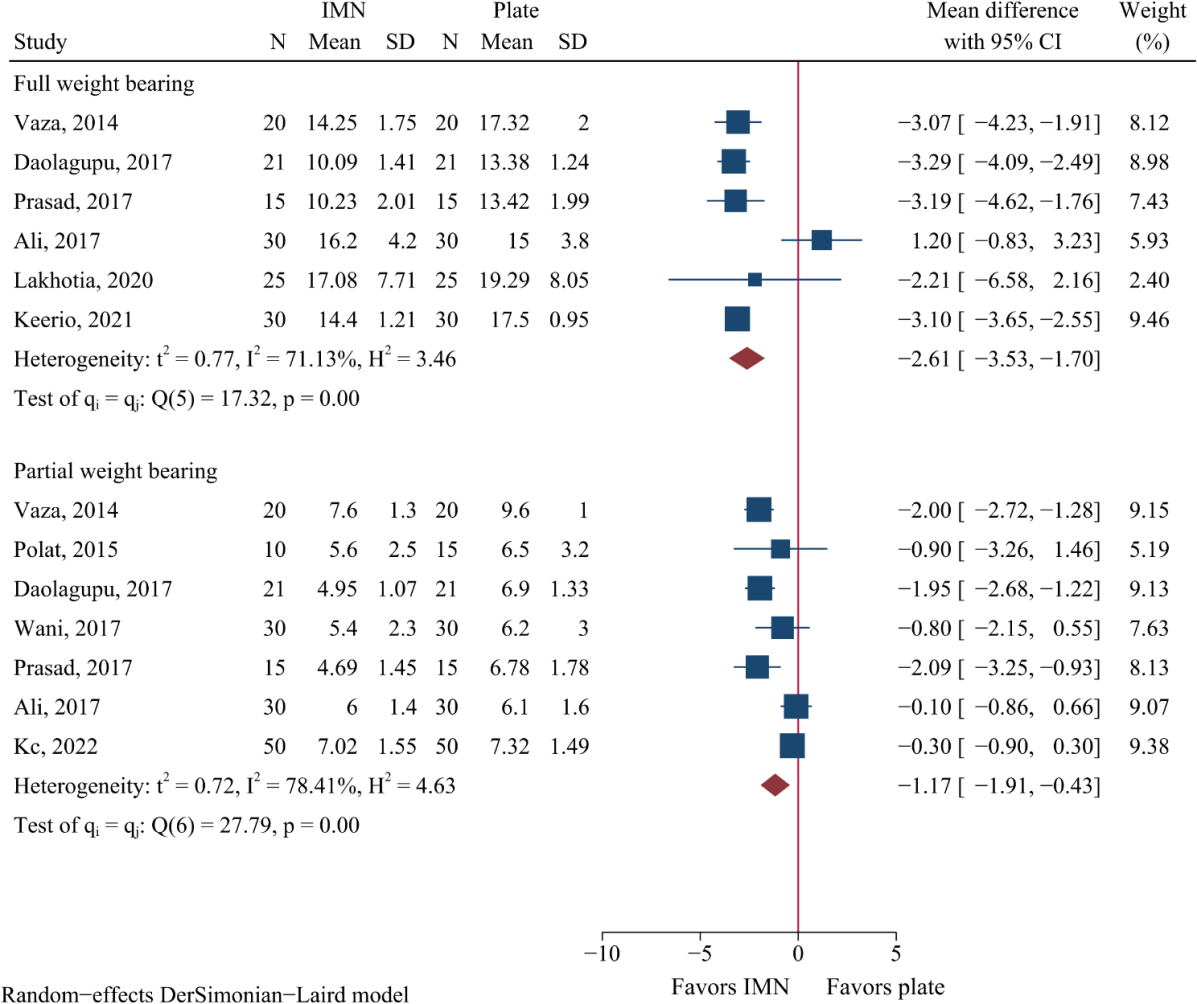
Meta-analysis of weight-bearing time in weeks comparing nail to plate fixation

As shown in Fig. 4, IMN conferred a 1.71-week shorter time to partial weight bearing (95%CI: 0.43 to 1.91, *P* = 0.002) and a 2.61-week shorter time to full weight bearing (95%CI: 1.70 to 3.53, *P* < 0.001) compared to plate fixation.

### Wound infection

**Fig. 5:**
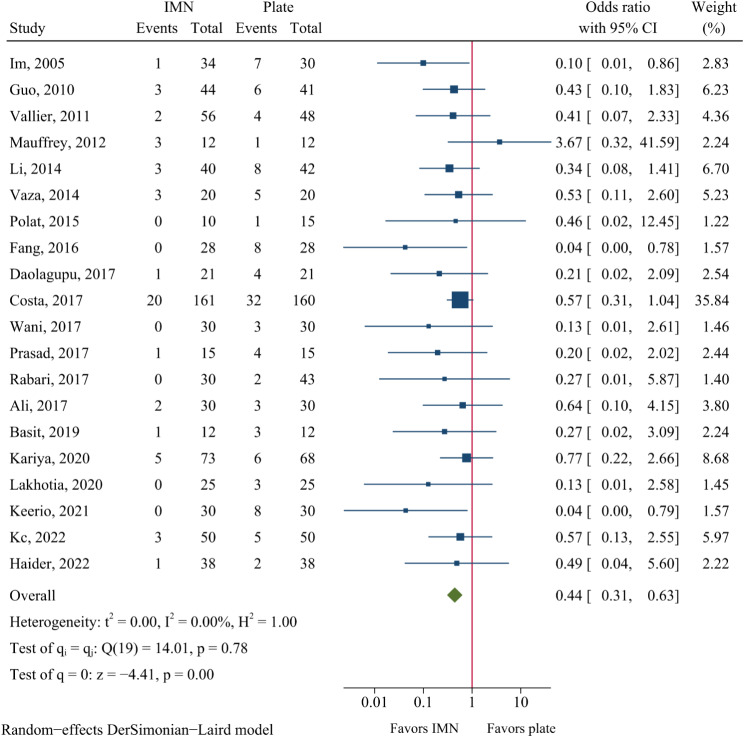
Meta-analysis of wound infection comparing nail to plate fixation

Wound infection was documented in 20 RCTs, which included 759 patients in the IMN group and 759 in the plate fixation group. The rate of wound infections was 6.5% and 15.2% in each group, respectively. Therefore, IMN was associated with a significantly reduced risk of wound infection compared to plate fixation (OR = 0.44, 95%CI: 0.31–0.63, *P* < 0.001, Fig. 5).

### Nonunion, malunion and delayed union

**Fig. 6:**
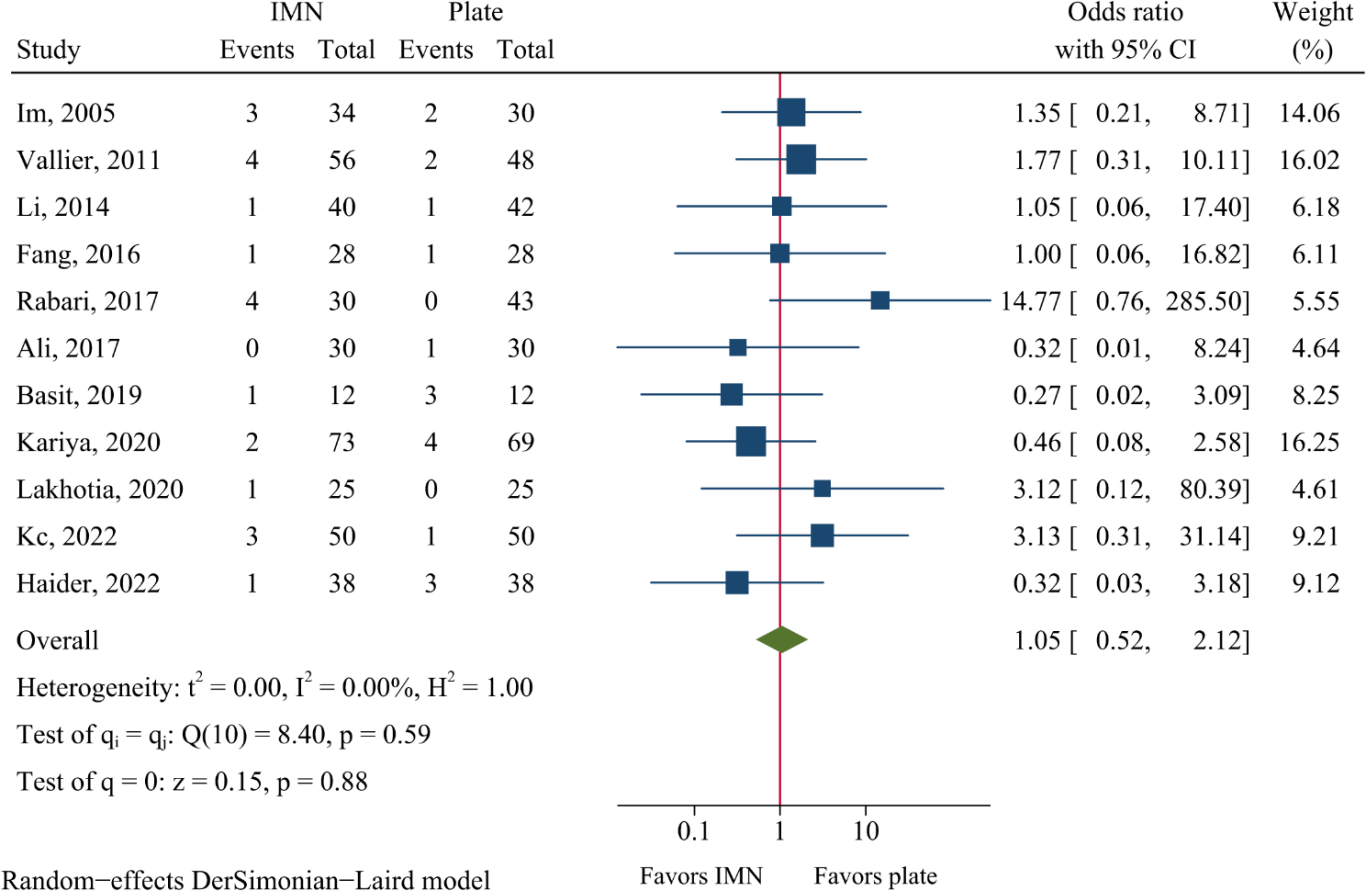
Meta-analysis of nonunion comparing nail to plate fixation

**Fig. 7:**
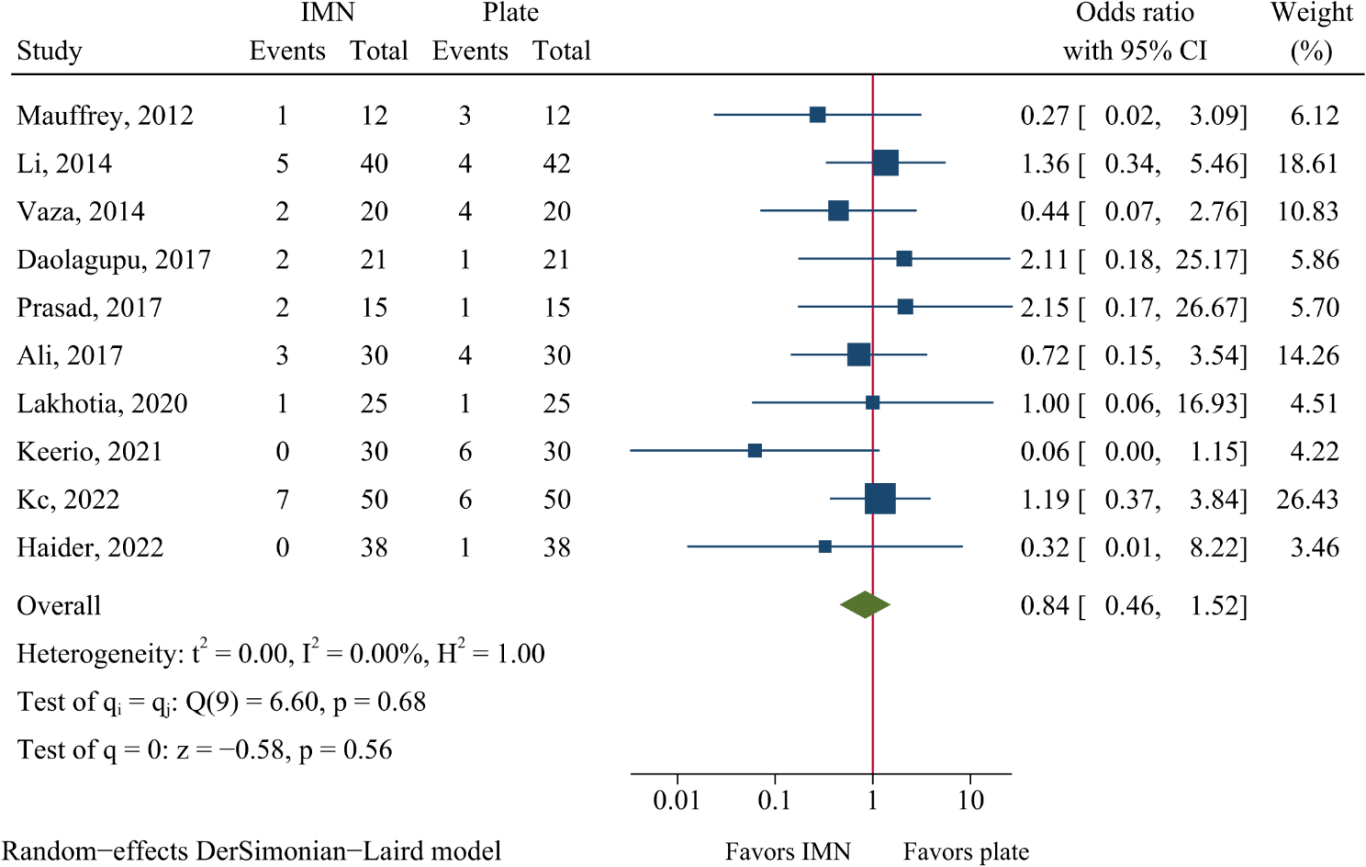
Meta-analysis of delayed union comparing nail to plate fixation

The rates of nonunion and delayed union were 5.0% (21/416) and 8.2 (23/281) in the IMN group, and 4.3% (18/415) and 11.0% (31/283) in the plate fixation group. The risk of nonunion and delayed union did not significantly differ between the IMN and plate fixation groups (OR = 1.05, 95%CI: 0.52–2.12, *P* = 0.883, Fig. 6; OR = 0.84, 95%CI: 0.46–1.52, *P* = 0.559, Fig. 7).

**Fig. 8:**
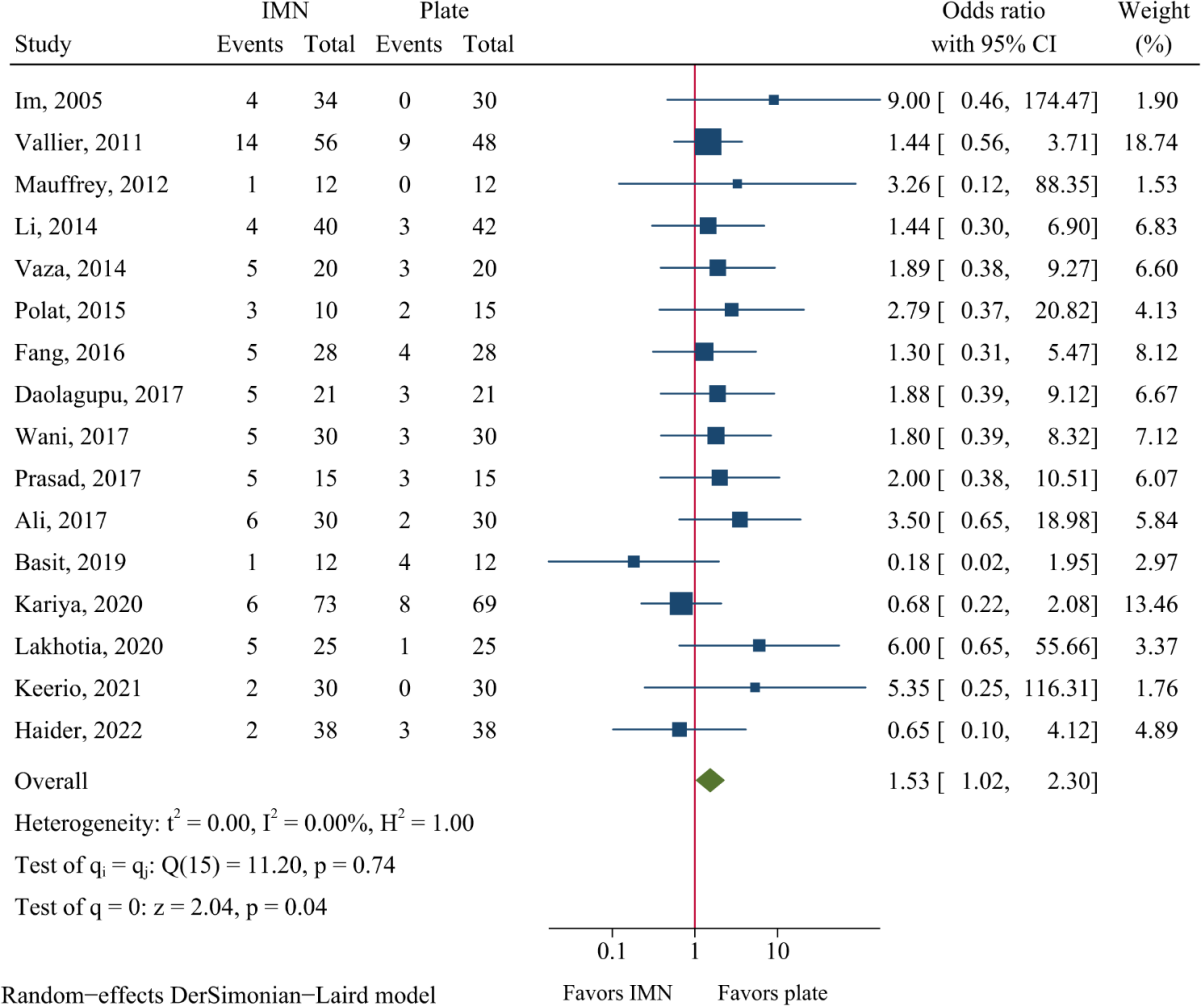
Meta-analysis of malunion comparing nail to plate fixation

In the IMN group, 15.4% (73/484) of patients developed malunion, which was higher than 10.3% (48/465) in the plate fixation group. Thus, IMN was associated with a higher risk of malunion compared to plate fixation (OR = 1.53, 95%CI: 1.02–2.30, *P* = 0.041, Fig. 8).

### Anterior knee pain

**Fig. 9:**
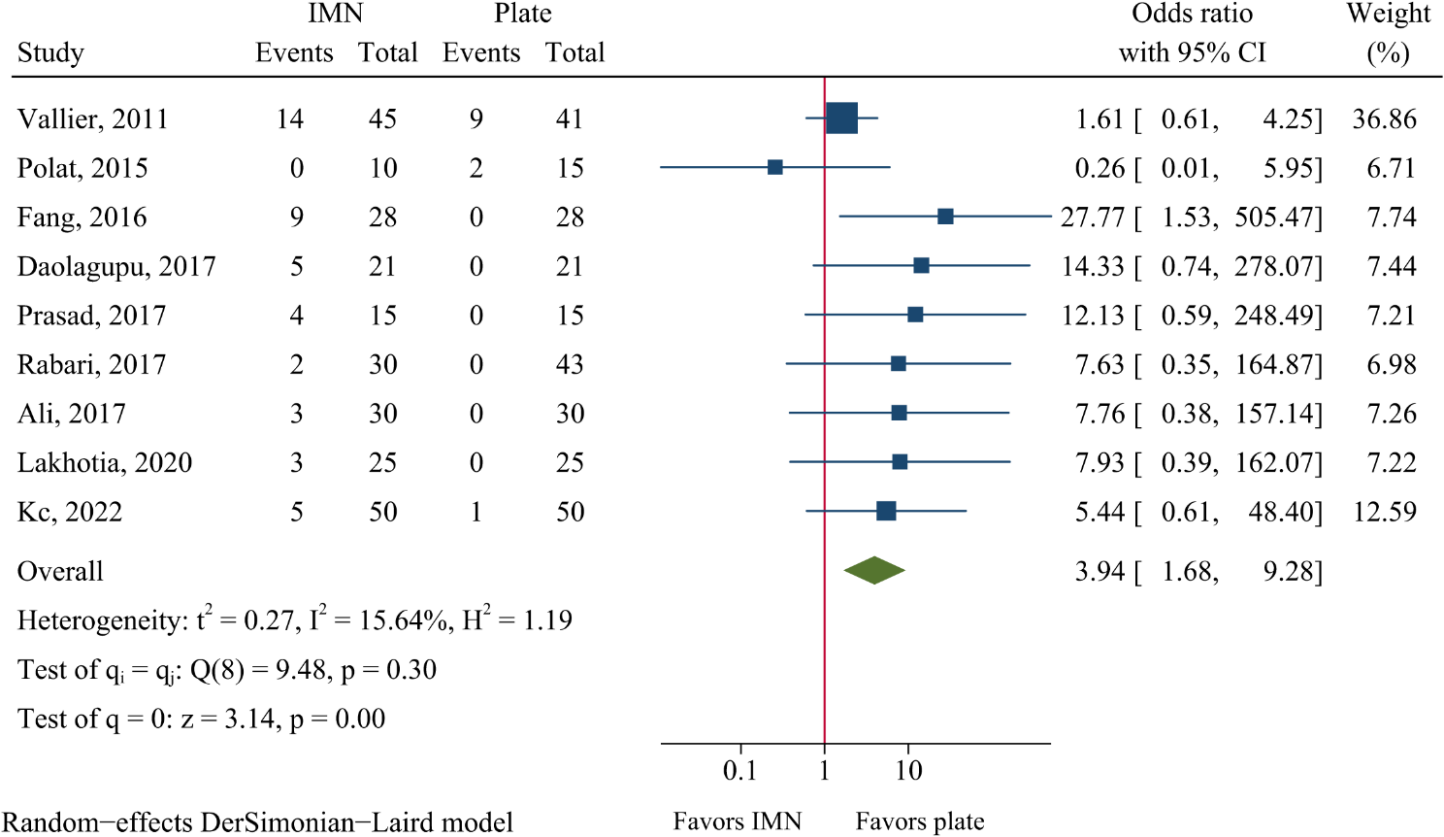
Meta-analysis of anterior knee pain comparing nail to plate fixation

There were 45 cases of anterior knee pain among 254 IMN-treated patients and only 12 cases among 268 plate-treated patients. The rate of anterior knee pain was significantly higher in the IMN group than in the plate fixation group (17.7% vs. 4.5%, OR = 3.94, 95%CI: 1.68–9.28, *P* = 0.002, Fig. 9).

### Secondary procedures

**Fig. 10:**
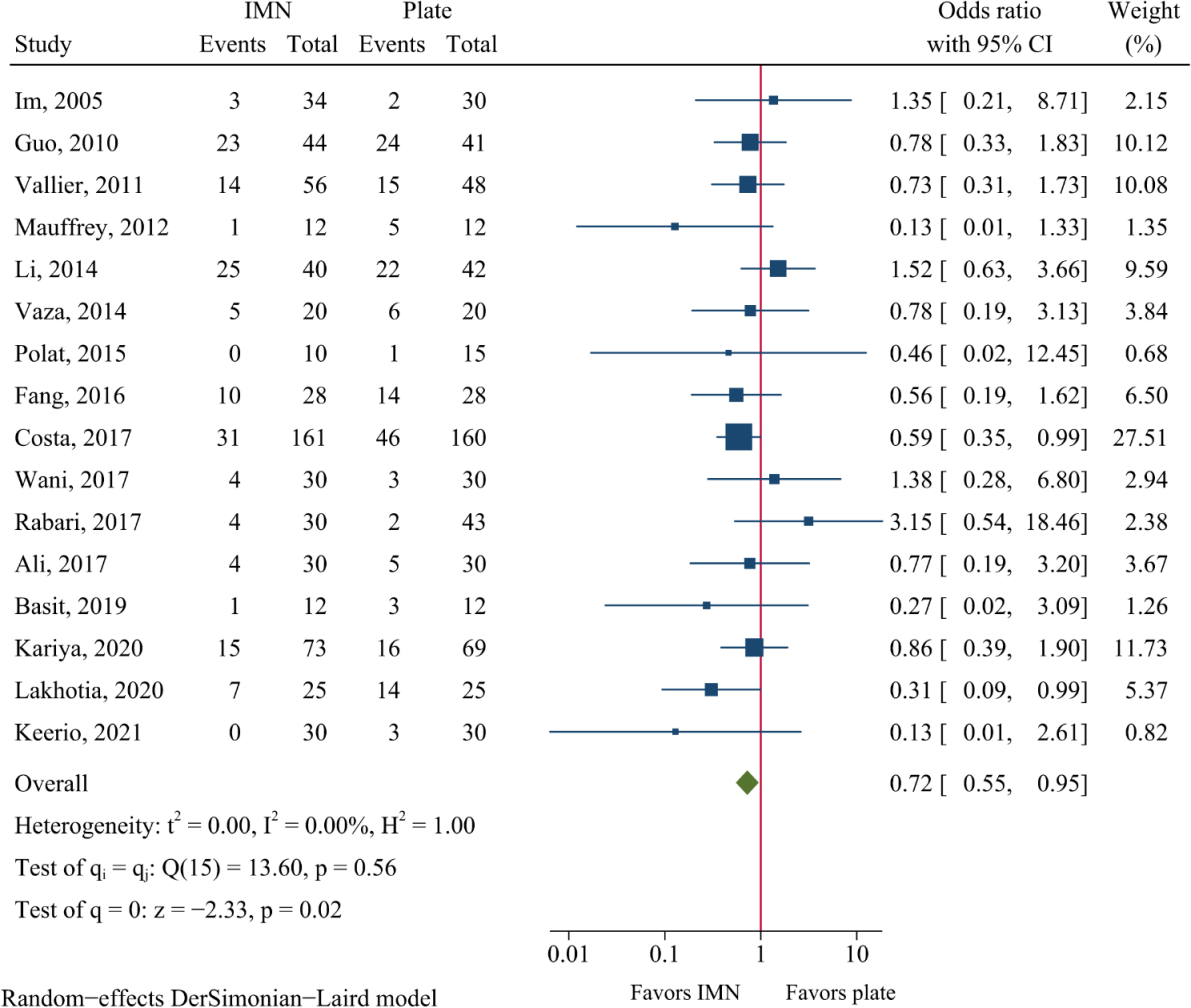
Meta-analysis of secondary procedures comparing nail to plate fixation

Secondary procedures were reported in 16 studies, including 635 patients in the IMN group and 635 patients in the plate fixation group. A secondary procedure was required in 23.1% of patients in the IMN group and 28.5% in the plate fixation group. A significantly lower risk of secondary procedures was observed in the IMN group compared to the plate fixation group (OR = 0.72, 95%CI: 0.55–0.95, *P* = 0.020, Fig. 10).

### Functional assessment

**Fig. 11:**
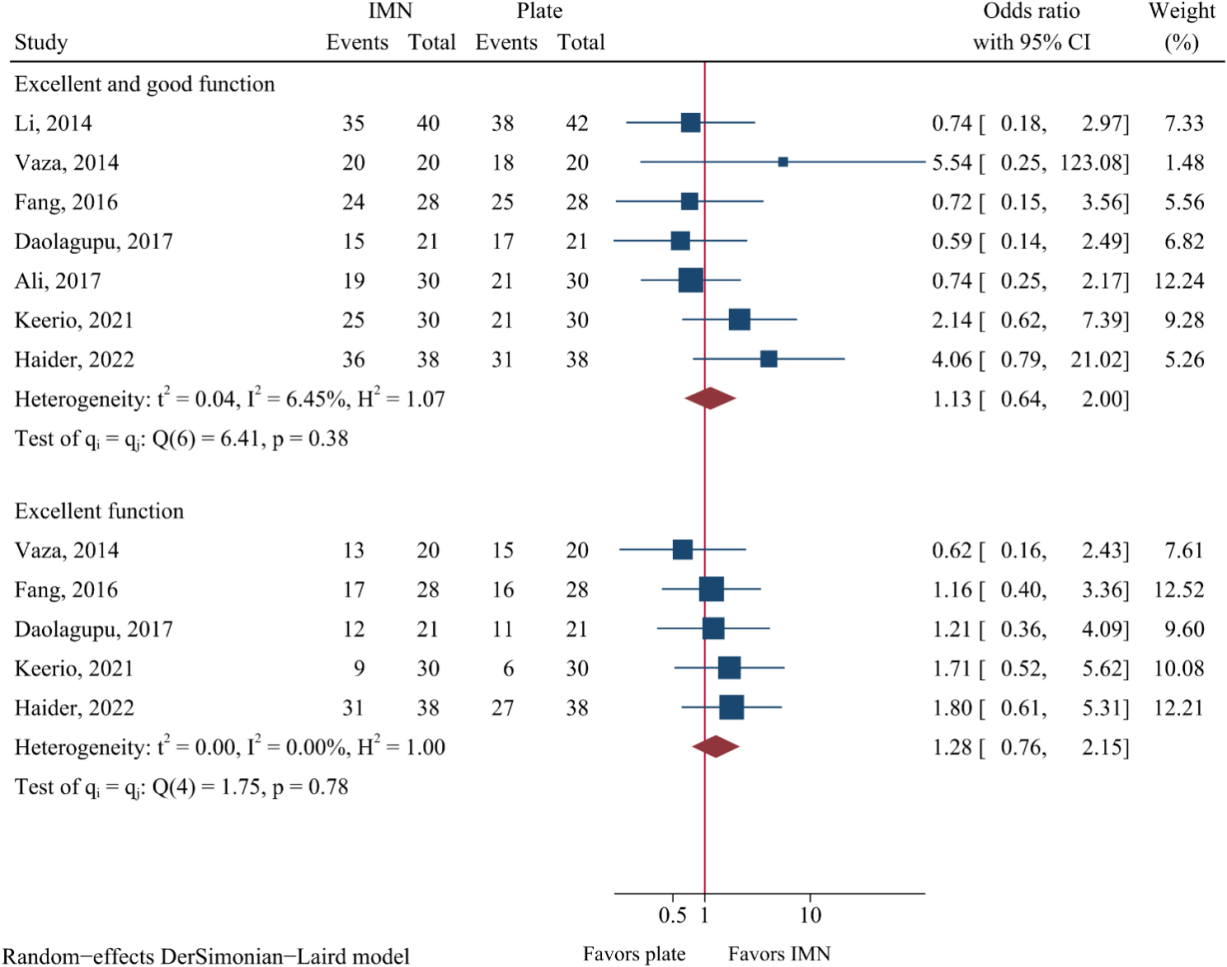
Meta-analysis of categorical functional outcomes comparing nail to plate fixation

There was no significant difference between both groups in functional assessment in terms of AOFAS scores (Additional file 3: Figure S2), DRI scores (Additional file 3: Figure S3), and FFI scores (Additional file 3: Figure S4). The functional outcome in several studies was further rated as an excellent, good, fair, or poor according to assessment scores such as AOFAS [17, 28, 39], OMAS [37, 43, 44], and MAS [30]. The proportion of patients with an excellent result was 59.9% (82/137) and 54.7% (75/137) in the IMN and plate fixation groups, respectively. The proportion of patients with excellent and good functional outcomes was 84.1% (174/207) and 81.8% (171/209), respectively. No significant difference in the proportions was observed between both groups (Excellent and good function: OR = 1.13, 95%CI: 0.64-2.00, *P* = 0.668; Excellent function: OR = 1.28, 95%CI: 0.76–2.15, *P* = 0.361; Fig. 11).

### Subgroup analysis

The results of further subgroup analyses stratified by plate fixation technique (MIPO, ORIF), AO classification (AO 42, AO 43), and inclusion of open fractures were summarized in Additional file 2: Table S3. Among all subgroups, IMN significantly reduced the risk of wound infection and shortened surgery time compared to plate fixation. The incidence of secondary procedures was reduced at a marginal significance (OR = 0.73, 95%CI: 0.53-1.00, *P* = 0.053) when comparing IMN to MIPO, and was significantly reduced when comparing IMN to plate fixation in the subgroup excluding open fractures (OR = 0.67, 95%CI 0.47–0.94, *P* = 0.021). In the subgroup including open fractures, the risk of malunion was significantly higher in IMN than plate fixation (OR = 1.80, 95%CI: 1.01–3.21, *P* = 0.048). An increased risk of anterior knee pain was observed in MIPO technique, AO 43 factures, and the subgroup excluding open fractures, but not in AO 43 fractures or the subgroup including open fractures.

### Sensitivity analysis and meta-regression analysis

Sensitivity analysis revealed that omitting a single study did not significantly affect the pooled results. Several baseline characteristics, including percentages of male, closed fractures, mean age and publication year, were analyzed. The percentage of concomitant fibula fractures was not analyzed as fewer then 10 studies reported this data. Meta-regression analysis demonstrated that none of these features were significantly associated with the effect size of the pooled analysis, suggesting that they were not modulating factors for the efficacy comparison between IMN and plate fixation (Additional file 2: Table S4).

### Publication bias

Egger’s test suggested no obvious publication bias in most of the analyzed outcomes but implied potential publication bias in wound infection and anterior knee pain (Additional file 2: Table S5). After adding 5 imputed studies in the analysis of wound infection, the OR estimate increased from 0.44 to 0.55 (95%CI: 0.38–0.79, Additional file 3: Figure S5), which was still statistical significant. After including 3 imputed studies in the analysis of anterior knee pain, the OR estimate decreased from 3.94 to 2.57 (95%CI: 1.06–6.26, Additional file 3: Figure S6), which remained statistically significant.

## Discussion

Distal tibia is susceptible to injury due to its low soft tissue coverage and insufficient blood supply, making the treatment and management of distal tibia fractures challenging. Two surgical modalities, IMN and plate fixation, are commonly used in clinical practice, but the optimal surgical strategy remains controversial. This meta-analysis included 1528 patients with distal tibia fractures from 20 RCTs to compare the functional outcomes and complication rates between IMN and plate fixation. We found that IMN had several advantages over plate fixation, including shortening surgery time, union time, and weight-bearing time, as well as reducing the risk of wound infections and secondary procedures. Both modalities achieved comparable functional outcomes and had similar rates of nonunion and delayed union. However, the major drawbacks of IMN were increased rates of anterior knee pain and malunion.

Wound infection, particularly deep infection, is one of the major complications after distal tibia fracture surgery and is associated with re-intervention, delayed healing, soft tissue scarring, and prolonged use of antibiotics [45]. Plate fixation requires more stripping of soft tissues and periosteum, leading to a higher risk of wound infections [46]. The wound infection rate was 6.5% in the IMN group and 15.2% in the plate fixation group, showing a statistically significant difference. Furthermore, deep infections appear to be more prevalent in plate fixation using the ORIF technique than the MIPO technique. Keerio et al. observed eight deep infections in the ORIF group but none in the IMN group [43]. Vallier et al. reported that 2 out of 56 IMN-treated patients suffered deep infection, compared to 4 out of 48 ORIF-treated patients [32]. Thus, ORIF plating had a higher deep infection rate compared to IMN (12.0% vs. 1.67%, *P* = 0.036). In contrast, the rates were 1.4% (3/221) and 3.6% (8/223) with no significant difference in the comparison of IMN vs. MIPO.

Our meta-analysis suggested that IMN shortened the time to partial weight bearing by 1.17 weeks, the time to full partial weight bearing by 2.61 weeks, and the time to union by 1.56 weeks compared to plate fixation. However, these results should be interpreted with caution. Firstly, there was substantial heterogeneity in the meta-analysis. Secondly, the assessment of time to radiographic union is challenging due to the lack of an established gold standard and significant inter-observer disagreement [47]. Nonetheless, we observed only a 1-week difference of union time when comparing IMN to MIPO but a 5-week difference when comparing IMN to ORIF. Whether these differences of nearly 2 weeks could translate into clinical benefit is uncertain and needs to be ascertained by future studies.

Anterior knee pain is a major complication of IMN, with an incidence of 17.7%, which may influence the choice between IMN and plate fixation. The pain persisted in up to 58% of patients even after implant removal [48, 49]. The incidence of knee pain did not differ between the two IMN approaches, i.e. suprapatellar (SP) and infrapatellar (IP) [50]. However, recent trials suggested that the SP approach could more effectively relieve anterior knee pain than the IP approach. Lu et al. performed a retrospective analysis to compare SP to IP in IMN-treated patients with distal tibia fractures [51]. They found lower levels of knee pain, higher AOFAS scores, and a reduced rate of fracture deformity in the SP approach compared to the IP approach. Similar results, including less pain and lower rate of malalignment, were also observed in other retrospective studies [52, 53]. Furthermore, several RCTs demonstrated significant improvement in knee function and a reduction in anterior knee pain when comparing SP to IP [54–56]. Therefore, SP approach may be the preferred choice for distal tibia fracture patients receiving IMN treatment [57].

The present meta-analysis has several strengths compared to previous ones [6, 7, 9, 58–61]. We compared efficacy across various aspects including surgery time, union time, weight-bearing time, functional outcomes, and complications. The functional outcomes encompassed several scoring systems and were further categorized into good and excellent outcomes. Therefore, the efficacy of IMN and plate fixation can be more comprehensively compared, aiding in the decision-making for surgical treatments. Secondly, our study, focusing on RCTs, included the most eligible trials with the largest sample size (*n* = 1528), providing more statistical power to detect differences. We found that the IMN group had a significantly lower rate of secondary procedures, a finding not reported by previous meta-analyses [6, 58, 59]. Thirdly, we performed more detailed subgroup analyses according to plate fixation technique (MIPO, ORIF), AO classification (AO 42, AO 43), and the inclusion of open fractures. Additionally, we performed meta-regression analysis to identify potential modulators of efficacy, although none reached statistical significance. We also analyzed functional outcomes as categorical outcomes, showing no difference between both groups in the percentage of patients achieving an excellent outcome and in the percentage of patients obtaining an excellent and good outcome. These analyses, not conducted in previous meta-analyses, would definitely aid in personalized surgical decision-making in patients under difference circumstances.

Several limitations of our study should be noticed. Firstly, most included trials had a small sample size, preventing further analysis on subgroups stratified by baseline characteristics of patients and fractures. Secondly, the characteristics of fractures such as open fractures, AO classification, fibula fractures, and fibula fixation are diverse, which may introduce heterogeneity. Thirdly, the functional outcomes were assessed by using different scores including AOFAS, OMAS, MAS, DRI and FFI, preventing a pooled analysis of quantitative functional outcomes. We could only analyze each score by pooling a few studies together. A uniform and standardized scoring system to report functional outcomes is required.

## Conclusions

The present meta-analysis of RCTs underscores the comparable efficacy of IMN and plate fixation in treating distal tibia fracture, while also highlighting their respective advantages and disadvantages. IMN accelerates union and weight bearing, shortens surgery time, and reduces wound infections and the need for secondary procedures. However, it is associated with a higher rate of anterior knee pain and malunion. The decision on the optimal surgical modality should consider the patient’s specific circumstances and characteristics. For young, healthy patients with minimal anterior tibia soft tissue damage and a lower risk of infection, plate fixation, particularly using the MIPO technique, may be a favorable choice to reduce the incidence of anterior knee pain and malunion. Conversely, for older patients, those with comorbidities, or those with severely injured soft tissues, IMN can be recommended to mitigate the risk of infections and secondary procedures.

### Electronic supplementary material

Below is the link to the electronic supplementary material.

Supplementary Material 1

Supplementary Material 2

## Data Availability

Data is provided within the manuscript or supplementary information files.

## Abbreviations

AOFAS: American Orthopaedic Foot and Ankle surgery
DRI: Disability Rating Index
FFI: Foot Function Index
IMN: intramedullary nail
MAS: Mazur ankle score
MIPO: minimally invasive percutaneous plate osteosynthesis
OMAS: Olreud Molander Ankle Scores
OR: odds ratio
ORIF: Open reduction and internal fixation
RCT: Randomized controlled trial
WMD: Weighted mean difference
