# Supplementary material for: Efficacy comparison between intramedullary nail fixation and plate fixation in distal tibia fractures: a meta-analysis of randomized controlled trials
Source: J Orthop Surg Res. 2024 Jul 12;19:403. doi: 10.1186/s13018-024-04900-y (PMC11241967; doi:10.1186/s13018-024-04900-y)
Supplement: Supplementary file 1 — Supplementary Material 1 [file 13018_2024_4900_MOESM1_ESM.docx]

Table S1. Search strategy for each database.

| Literature database | Search strategy |
| --- | --- |
| PubMed | (‘Tibial Fractures’[MeSH] OR ‘tibial fractures’[Title/Abstract] OR ‘tibia*’[Title/Abstract]) AND (‘Fracture Fixation, Intramedullary’[MeSH] OR ‘intramedullary nail*’[Title/Abstract] OR ‘intramedullary fixation’[Title/Abstract] OR ‘interlocking nail*’[Title/Abstract]) AND (‘Randomized Controlled Trial’[MeSH] OR ‘randomized’[Title/Abstract] OR ‘randomised’[Title/Abstract] OR ‘randomization’[Title/Abstract]) |
| Web of Science | (‘tibial fractures’ OR ‘distal tibia*’ OR ‘tibia*’) AND (‘intramedullary nail*’ OR ‘intramedullary fixation’ OR ‘interlocking nail*’) AND (‘randomized’ OR ‘randomised’ OR ‘randomization’) |
| EMBASE | (‘tibial fractures’ OR ‘distal tibia*’ OR ‘tibia*’) AND (‘intramedullary nail*’ OR ‘intramedullary fixation’ OR ‘interlocking nail*’) AND (‘randomized’ OR ‘randomised’ OR ‘randomization’) |
| Clinicaltrials.gov | (‘tibial fractures’ OR ‘distal tibia*’ OR ‘tibia*’) AND (‘intramedullary nail*’ OR ‘intramedullary fixation’ OR ‘interlocking nail*’) AND (‘randomized’ OR ‘randomised’ OR ‘randomization’) |
| Cochrane Library | (‘tibial fractures’ OR ‘distal tibia*’ OR ‘tibia*’) AND (‘intramedullary nail*’ OR ‘intramedullary fixation’ OR ‘interlocking nail*’) AND (‘randomized’ OR ‘randomised’ OR ‘randomization’) |

Table S2. Methodology assessment using the Cochrane Collaboration’s tool for assessing risk of bias.

| Study | Random sequence generation (selection bias) | Allocation concealment (selection bias) | Blinding of participants and personnel (performance bias) | Blinding of outcome assessment (detection bias) | Incomplete outcome data (attrition bias) | Selective reporting (reporting bias) | Other bias |
| --- | --- | --- | --- | --- | --- | --- | --- |
| Im, 2005 | Low | Low | Unclear | Unclear | Low | Low | Low |
| Guo, 2010 | Unclear | Unclear | Unclear | Unclear | Low | Low | Low |
| Vallier, 2011 | Low | Low | Unclear | Unclear | Low | Low | Low |
| Mauffrey, 2012 | Low | Low | Unclear | Low | Low | Low | Low |
| Li, 2014 | Low | Low | Unclear | Unclear | Low | Low | Low |
| Vaza, 2014 | Unclear | Unclear | Unclear | Unclear | Low | Low | Low |
| Polat, 2015 | Low | Low | Unclear | Unclear | Low | Low | Low |
| Fang, 2016 | Low | Low | Unclear | Low | Low | Low | Low |
| Daolagupu, 2017 | Low | Low | Unclear | Unclear | Low | Low | Low |
| Costa, 2017 | Low | Low | Unclear | Low | Low | Low | Low |
| Wani, 2017 | Low | Unclear | Unclear | Unclear | Low | Low | Low |
| Rabari, 2017 | Unclear | Unclear | Unclear | Unclear | Low | Low | Low |
| Ali, 2017 | Low | Unclear | Unclear | Unclear | Low | Low | Low |
| Prasad, 2017 | Low | Unclear | Unclear | Unclear | Low | Low | Low |
| Basit, 2019 | Low | Low | Unclear | Low | Low | Low | Low |
| Kariya, 2020 | Low | Low | Unclear | Unclear | Low | Low | Low |
| Lakhotia, 2020 | Low | Unclear | Unclear | Unclear | High | Low | Low |
| Keerio, 2021 | Low | Unclear | Unclear | Unclear | Low | Low | Low |
| Kc, 2022 | Low | Unclear | Unclear | Unclear | Low | Low | Low |
| Haider, 2022 | Low | Unclear | Unclear | Unclear | Low | Low | Low |

Table S3. Results of analysis comparing IMN to plate fixation technique (MIPO, ORFI), AO classification (AO 42, AO 43), and inclusion of open fractures.

| Outcome | No. of study | No. of patients | I^2^, % | Effect size (95%CI) | P |
| --- | --- | --- | --- | --- | --- |
| **Continuous variables** |  |  |  | **Mean difference** |  |
| Surgery time, minutes |  |  |  |  |  |
| MIPO | 8 | 335/331 | 88.7 | -10.32 (-16.39, -4.24) | <0.001 |
| ORIF | 1 | - | - | - | - |
| AO 42 | 4 | 151/154 | 77.2 | -12.70 (-22.26, -3.15) | 0.009 |
| AO 43 | 6 | 186/177 | 88.7 | -9.57 (-15.86, -3.28) | 0.003 |
| Including open fractures | 4 | 122/120 | 63.0 | -15.52 (-22.81, -8.23) | <0.001 |
| Excluding open fractures | 6 | 213/211 | 89.7 | -7.84 (-14.42, -1.25) | 0.020 |
| Radiation time, minutes |  |  |  |  |  |
| MIPO | 5 | 205/203 | 94.5 | -0.79 (-1.31, -0.27) | 0.003 |
| ORIF | 0 | - | - | - | - |
| AO 42 | 3 | 111/112 | 94.8 | -0.12 (-1.44, 1.21) | 0.862 |
| AO 43 | 2 | 94/91 | 19.4 | -0.95 (-1.16, -0.74) | <0.001 |
| Including open fractures | 1 | - | - | - | - |
| Excluding open fractures | 4 | 177/175 | 94.8 | -0.60 (-1.16, -0.04) | 0.037 |
| Union time, weeks |  |  |  |  |  |
| MIPO | 12 | 367/382 | 59.4 | -1.02 (-1.67, -0.37) | 0.002 |
| ORIF | 2 | 64/60 | 0 | -5.15 (-5.74, -4.55) | <0.001 |
| AO 42 | 4 | 114/119 | 0.6 | -0.43 (-1.03, 0.16) | 0.153 |
| AO 43 | 9 | 287/293 | 95.4 | -2.18 (-4.01, -0.35) | 0.020 |
| Including open fractures | 4 | 138/134 | 0 | 0.11 (-1.06, 1.29) | 0.849 |
| Excluding open fractures | 10 | 293/308 | 95.0 | -1.86 (-3.31, -0.41) | 0.012 |
| Partial weight-bearing time, weeks |  |  |  |  |  |
| MIPO | 6 | 156/161 | 75.1 | -1.00 (-1.79, -0.21) | 0.013 |
| ORIF | 0 | - | - | - | - |
| AO 42 | 2 | 40/45 | 0 | -0.83 (-2.00, 0.35) | 0.168 |
| AO 43 | 4 | 106/106 | 84.4 | -1.54 (-2.51, -0.58) | 0.002 |
| Including open fractures | 2 | 50/50 | 92.1 | -1.05 (-2.92, 0.81) | 0.267 |
| Excluding open fractures | 5 | 126/131 | 73.6 | -1.23 (-2.13, -0.33) | 0.008 |
| Full weight-bearing time, weeks |  |  |  |  |  |
| MIPO | 4 | 91/91 | 82.1 | -1.98 (-3.98, 0.02) | 0.052 |
| ORIF | 1 | - | - | - | - |
| AO 42 | 0 | - | - | - | - |
| AO 43 | 5 | 111/111 | 0 | -3.14 (-3.55, -2.74) | <0.001 |
| Including open fractures | 2 | 50/50 | 92.2 | -1.02 (-5.20, 3.16) | 0.633 |
| Excluding open fractures | 4 | 91/91 | 0 | -3.15 (-3.59, -2.72) | <0.001 |
| AOFAS score |  |  |  |  |  |
| MIPO | 6 | 250/243 | 0 | 1.01 (-0.25, 2.28) | 0.117 |
| ORIF | 0 | - | - | - | - |
| AO 42 | 2 | 101/97 | 0 | 1.26 (-0.45, 2.96) | 0.148 |
| AO 43 | 3 | 119/116 | 0 | 1.30 (-0.82, 3.41) | 0.229 |
| Including open fractures | 2 | 58/58 | 0 | -0.88 (-3.59, -2.72) | 0.560 |
| Excluding open fractures | 4 | 192/185 | 0 | 1.46 (0.05, 2.88) | 0.042 |
| DRI score |  |  |  |  |  |
| MIPO | 2 | 154/152 | 0 | -3.96 (-8.49, 0.56) | 0.086 |
| ORIF | 0 | - | - | - | - |
| AO 42 | 0 | - | - | - | - |
| AO 43 | 1 | - | - | - | - |
| Including open fractures | 1 | - | - | - | - |
| Excluding open fractures | 1 | - | - | - | - |
| FFI score |  |  |  |  |  |
| MIPO | 2 | 40/45 | 0 | -1.22 (-6.79, 4.33) | 0.668 |
| ORIF | 1 | - | - | - | - |
| AO 42 | 3 | 85/86 | 6.5 | 1.13 (-3.68, 5.95) | 0.645 |
| AO 43 | 0 | - | - | - | - |
| Including open fractures | 1 | - | - | - | - |
| Excluding open fractures | 2 | 40/45 | 0 | -1.22 (-6.79, 4.33) | 0.668 |
| **Categorical variables** |  |  |  | **Odds ratio** |  |
| Wound infection |  |  |  |  |  |
| MIPO | 16 | 619/631 | 0 | 0.48 (0.32-0.71) | <0.001 |
| ORIF | 3 | 120/108 | 2.1 | 0.17 (0.05-0.60) | 0.006 |
| AO 42 | 7 | 262/257 | 0 | 0.37 (0.18-0.76) | 0.007 |
| AO 43 | 11 | 306/312 | 0 | 0.37 (0.20-0.69) | 0.002 |
| Including open fractures | 7 | 220/210 | 19.9 | 0.40 (0.18-0.88) | 0.023 |
| Excluding open fractures | 13 | 539/549 | 0 | 0.46 (0.30-0.70) | <0.001 |
| Nonunion |  |  |  |  |  |
| MIPO | 9 | 326/337 | 0 | 0.89 (0.39-2.05) | 0.783 |
| ORIF | 2 | 90/78 | 0 | 1.56 (0.44-5.57) | 0.492 |
| AO 42 | 5 | 222/212 | 0 | 1.04 (0.39-2.82) | 0.936 |
| AO 43 | 5 | 164/173 | 35.1 | 1.24 (0.34-4.52) | 0.742 |
| Including open fractures | 5 | 188/178 | 0 | 1.20 (0.43-3.31) | 0.729 |
| Excluding open fractures | 6 | 228/237 | 32.6 | 1.05 (0.32-3.47) | 0.939 |
| Delayed union |  |  |  |  |  |
| MIPO | 8 | 231/233 | 0 | 1.03 (0.54-1.98) | 0.926 |
| ORIF | 1 | - | - | - | - |
| AO 42 | 2 | 65/67 | 0 | 1.28 (0.37-4.46) | 0.700 |
| AO 43 | 7 | 186/186 | 0 | 0.74 (0.35-1.58) | 0.435 |
| Including open fractures | 4 | 102/104 | 0 | 0.73 (0.31-1.71) | 0.468 |
| Excluding open fractures | 6 | 179/179 | 0 | 0.96 (0.41-2.24) | 0.919 |
| Malunion |  |  |  |  |  |
| MIPO | 12 | 334/337 | 0 | 1.41 (0.87-2.29) | 0.166 |
| ORIF | 3 | 120/108 | 0 | 1.87 (0.79-4.43) | 0.155 |
| AO 42 | 7 | 262/257 | 0 | 1.40 (0.83-2.36) | 0.203 |
| AO 43 | 8 | 182/178 | 0 | 1.56 (0.76-3.20) | 0.226 |
| Including open fractures | 7 | 220/210 | 0 | 1.80 (1.01-3.21) | 0.048 |
| Excluding open fractures | 9 | 254/255 | 3.4 | 1.32 (0.73-2.38) | 0.359 |
| Anterior knee pain |  |  |  |  |  |
| MIPO | 8 | 209/227 | 0 | 6.69 (2.43-18.32) | <0.001 |
| ORIF | 1 | - | - | - | - |
| AO 42 | 4 | 108/109 | 48.2 | 2.73 (0.54-13.86) | 0.225 |
| AO 43 | 4 | 116/129 | 0 | 8.40 (2.15-32.81) | 0.002 |
| Including open fractures | 3 | 103/99 | 49.7 | 4.51 (0.76-26.80) | 0.098 |
| Excluding open fractures | 6 | 151/169 | 0 | 5.20 (1.64-16.51) | 0.005 |
| Secondary procedures |  |  |  |  |  |
| MIPO | 12 | 495/507 | 7.5 | 0.73 (0.53-1.00) | 0.053 |
| ORIF | 3 | 120/108 | 0 | 0.73 (0.34-1.55) | 0.408 |
| AO 42 | 7 | 262/257 | 0 | 0.80 (0.53-1.19) | 0.261 |
| AO 43 | 7 | 182/188 | 15.7 | 0.73 (0.37-1.45) | 0.370 |
| Including open fractures | 7 | 220/210 | 0 | 0.83 (0.53-1.30) | 0.408 |
| Excluding open fractures | 9 | 415/425 | 0 | 0.67 (0.47-0.94) | 0.021 |
| Excellent function |  |  |  |  |  |
| MIPO | 3 | 87/87 | 0 | 1.37 (0.72-2.61) | 0.334 |
| ORIF | 1 | - | - | - | - |
| AO 42 | 1 | - | - | - | - |
| AO 43 | 4 | 109/109 | 0 | 1.32 (0.72-2.40) | 0.370 |
| Including open fractures | 2 | 48/48 | 0 | 0.91 (0.40-2.12) | 0.835 |
| Excluding open fractures | 3 | 89/89 | 0 | 1.58 (0.81-3.07) | 1.82 |
| Excellent and good function |  |  |  |  |  |
| MIPO | 5 | 157/159 | 0 | 0.90 (0.48-1.67) | 0.734 |
| ORIF | 1 | - | - | - | - |
| AO 42 | 2 | 68/70 | 0 | 0.73 (0.26-2.09) | 0.556 |
| AO 43 | 4 | 109/109 | 21.7 | 1.84 (0.73-4.63) | 0.195 |
| Including open fractures | 4 | 118/120 | 0 | 0.82 (0.40-1.71) | 0.599 |
| Excluding open fractures | 3 | 89/89 | 39.3 | 1.67 (0.58-4.82) | 0.340 |

AOFAS: American Orthopaedic Foot and Ankle surgery; DRI: disability rating index; FFI: foot function index; MIPO: minimally invasive plate osteosynthesis; ORIF: open reduction and internal fixation.

Table S4. Results of meta-regression analysis.

| Modulators | Coefficient | Standard error | Lower limit | Upper limit | z | P | No. of studies |
| --- | --- | --- | --- | --- | --- | --- | --- |
| **Union time** |  |  |  |  |  |  |  |
| Percentage of males | -0.018 | 0.068 | -0.152 | 0.115 | -0.27 | 0.786 | 14 |
| Mean age | 0.140 | 0.200 | -0.253 | 0.533 | 0.7 | 0.486 | 14 |
| Percentage of closed fractures | -0.041 | 0.028 | -0.097 | 0.014 | -1.45 | 0.146 | 12 |
| Publication year | -0.236 | 0.135 | -0.501 | 0.029 | -1.75 | 0.081 | 14 |
| **Wound infection** |  |  |  |  |  |  |  |
| Percentage of males | -0.029 | 0.018 | -0.064 | 0.006 | -1.65 | 0.100 | 19 |
| Mean age | 0.061 | 0.047 | -0.031 | 0.153 | 1.30 | 0.193 | 19 |
| Percentage of closed fractures | 0.007 | 0.014 | -0.020 | 0.035 | 0.53 | 0.599 | 17 |
| Publication year | 0.028 | 0.051 | -0.072 | 0.129 | 0.55 | 0.580 | 20 |
| **Malunion** |  |  |  |  |  |  |  |
| Percentage of males | 0.003 | 0.022 | -0.040 | 0.046 | 0.14 | 0.887 | 15 |
| Mean age | 0.062 | 0.055 | -0.046 | 0.171 | 1.13 | 0.258 | 15 |
| Percentage of closed fractures | -0.008 | 0.013 | -0.034 | 0.017 | -0.65 | 0.516 | 14 |
| Publication year | -0.068 | 0.057 | -0.181 | 0.044 | -1.19 | 0.235 | 16 |
| **Nonunion** |  |  |  |  |  |  |  |
| Percentage of males | 0.012 | 0.026 | -0.040 | 0.064 | 0.46 | 0.646 | 11 |
| Mean age | 0.082 | 0.072 | -0.059 | 0.223 | 1.14 | 0.253 | 11 |
| Percentage of closed fractures | -0.017 | 0.024 | -0.064 | 0.030 | -0.71 | 0.476 | 10 |
| Publication year | -0.045 | 0.063 | -0.169 | 0.078 | -0.72 | 0.472 | 11 |
| **Secondary interventions** |  |  |  |  |  |  |  |
| Percentage of males | 0.021 | 0.014 | -0.006 | 0.048 | 1.53 | 0.127 | 15 |
| Mean age | 0.010 | 0.043 | -0.074 | 0.094 | 0.23 | 0.810 | 15 |
| Percentage of closed fractures | -0.009 | 0.009 | -0.027 | 0.009 | -1.02 | 0.306 | 14 |
| Publication year | -0.036 | 0.039 | -0.113 | 0.040 | -0.93 | 0.352 | 16 |

Table S5. Egger’s test for publication bias.

| Outcome | z | P |
| --- | --- | --- |
| Surgery time | -0.47 | 0.640 |
| Union time | 0.20 | 0.841 |
| Partial weight bearing time | -0.05 | 0.963 |
| Full weight bearing time | 1.58 | 0.115 |
| AOFAS score | -0.90 | 0.370 |
| Wound infection | -1.92 | 0.055 |
| Nonunion | 0.49 | 0.622 |
| Delayed union | -1.16 | 0.247 |
| Malunion | 1.33 | 0.185 |
| Anterior knee pain | 1.89 | 0.059 |
| Secondary procedures | -0.42 | 0.671 |
| Excellent function | -0.92 | 0.359 |
| Excellent and good function | 0.66 | 0.509 |
