# Supplementary material for: Efficacy comparison between intramedullary nail fixation and plate fixation in distal tibia fractures: a meta-analysis of randomized controlled trials
Source: J Orthop Surg Res. 2024 Jul 12;19:403. doi: 10.1186/s13018-024-04900-y (PMC11241967; doi:10.1186/s13018-024-04900-y)
Supplement: Supplementary file 2 — Supplementary Material 2 [file 13018_2024_4900_MOESM2_ESM.docx]

Figure S1. Meta-anlaysis of radiation time comparing nail to plate fixation.

Figure S2. Meta-anlaysis of AOFAS scores comparing nail to plate fixation.

Figure S3. Meta-anlaysis of DRI scores comparing nail to plate fixation.

Figure S4. Meta-anlaysis of FFI scores comparing nail to plate fixation.

Figure S5. Funnel plot of trim-and-fill analysis of wound infection.

Figure S6. Funnel plot of trim-and-fill analysis of anterior knee pain.
